# Occurrence and genetic diversity of the zoonotic rat hepatitis E virus in small mammal species, Spain

**DOI:** 10.1186/s13567-025-01492-1

**Published:** 2025-03-25

**Authors:** Javier Caballero-Gómez, Tomás Fajardo-Alonso, Lucia Rios-Muñoz, Raúl Cuadrado-Matías, Aitor Somoano, Rosario Panadero, María Casares-Jiménez, Ignacio García-Bocanegra, Laura Ruiz, Adrián Beato-Benítez, Francisco Ruiz-Fons, Débora Jiménez-Martín, Alberto Espí, Ana del Cerro, Remigio Martínez, Mario Frías, Antonio Rivero-Juárez, Antonio Rivero

**Affiliations:** 1 Grupo de Virología Clínica y Zoonosis, Unidad de Enfermedades Infecciosas, Instituto Maimónides de Investigación Biomédica de Córdoba (IMIBIC), Hospital Universitario Reina Sofía, Universidad de Córdoba, Córdoba, Spain; 2 CIBERINFEC, ISCIII-CIBER de Enfermedades Infecciosas, Instituto de Salud Carlos III, Madrid, Spain; 3 Departamento de Sanidad Animal, Grupo de Investigación en Sanidad Animal y Zoonosis (GISAZ), UIC Zoonosis y Enfermedades Emergentes ENZOEM, Facultad de Veterinaria, Universidad de Córdoba, Córdoba, Spain; 4 Grupo de Sanidad y Biotecnología (SaBio), Instituto de Investigación en Recursos Cinegéticos IREC (CSIC-UCLM-JCCM), Ciudad Real, Spain; 5 Área de Sanidad Animal, Servicio Regional de Investigación y Desarrollo Agroalimentario (SERIDA), Asturias, Spain; 6 Departamento de Patología Animal. Grupo Investigación en Sanidad Animal:Galicia (INVESAGA), Facultad de Veterinaria, Universidad de Santiago de Compostela, Lugo, Spain

**Keywords:** *Rocahepevirus ratti*, zoonoses, emerging, host range, hepeviruses, surveillance

## Abstract

Rat hepatitis E virus (ratHEV-C1) is a newly zoonotic and globally distributed virus of increasing public health concern. Although rats of the genus *Rattus* are the main reservoir of this emerging virus, the role of other small mammals in the epidemiology of ratHEV-C1 in Europe is unknown as large-scale survey studies specifically assessing the circulation of ratHEV-C1 in these species have not yet been conducted. In this study, we examined active ratHEV-C1 infection in 514 animals from seven different small mammal species sampled throughout Spain. The presence of viral RNA was evaluated for ratHEV-C1 infection using RT-qPCRs in liver tissue. Sequencing and analysis of ratHEV-C1 shedding in faeces were carried out in positive animals. Viral RNA was detected in 15 individuals (mean Ct value: 34.5), supposing a frequency of 2.9% (95% CI: 1.8–4.8). We confirmed ratHEV infection in house mice (14.5%; 11/76), common voles (0.7%; 2/288) and garden dormice (25.0%; 2/8) and viral shedding in faeces in 36.4% (4/11) of positive house mice. Infected animals were detected in urban areas, small ruminant farms and rural areas throughout Spain. Our sequences showed a considerable genetic diversity, and they were closely related with ratHEV-C1 strains detected in human and rats from Spain, Germany and Hungary. Our study confirms that ratHEV is not limited to *Rattus* genus, identifying other rodents’ species as potential host of ratHEV-C1 in Europe. Our results highlight the importance of continued surveillance in animals to fully understand the dynamics of ratHEV-C1 and its impact on public health.

**Supplementary Information:**

The online version contains supplementary material available at 10.1186/s13567-025-01492-1.

## Introduction

*Rocahepevirus* (Hepeviridae family) is an emerging genus of global concern, encompassing a growing diversity of single-stranded RNA viruses, including two recognized genotypes (C1 and C2) and several putative genotypes (C3–C5) [1]. While ferrets (*Mustela putorius*), field mice (*Apodemus* sp.), the Pere David’s vole (*Eothenomys melanogaster*) and other voles (*Microtus spp.*) serve as the primary hosts of genotypes C2 to C5, respectively, rats of the genus *Rattus* are the main reservoirs of the *Rocahepevirus ratti* genotype C1 (rat hepatitis E virus; henceforth, ratHEV-C1) [1]. Initially, these were considered the only animal hosts of ratHEV-C1. However, the host range of this genotype has expanded in recent years, and ratHEV-C1 is now recognized as an emerging zoonotic virus of increasing public health concern globally [1–3]. In 2018, the first human case of ratHEV-C1 infection was reported in Hong Kong [4]. Since then, an increasing number of acute and/or chronic hepatitis cases have been reported not only in Asia [5], but also in America (in a patient traveling from Africa) [6] and Europe [7–10]. Besides, similar ratHEV-C1 strains to those found in rats and humans have also been detected in other small mammal species from Asia, such as house mice (*Mus musculus*), the greater bandicoot (*Bandicota indica*) and the Asian musk shrew (*Suncus murinus*) [3, 11]. In Europe, endemic circulation of ratHEV-C1 has been observed in *Rattus* populations [12] and phylogenetically related viral strains from the *Rocahepevirus* genus, including those from genotype 5 and previously unclassified strains, have been detected in an increasing number of other rodent species [1]. However, the role of small mammals other than *Rattus* in the epidemiology of ratHEV-C1 in Europe is unknown as large-scale survey studies specifically assessing the circulation of ratHEV-C1 in these species have not yet been conducted.

## Materials and methods

### Sampling

We designed a retrospective nationwide cross-sectional study in Spain. Population of several species of small mammals were sampled between 2012 and 2023 with the main aim to assess the prevalence of infection of ratHEV in these species (Additional file 1). Liver from all animals and, whenever possible, faeces from rectum or distant colon were collected and stored at −20 °C until analyses. Whenever possible, epidemiological information of each sampled individual, such as its species (based on external morphology), age, sex, habitat, sampling date and sampling location, was recorded (Table 1).

**Table 1:** **Demographic data of small mammals sampled in Spain**

Variable	Categories	No (%)
Species	*Microtus arvalis*	288 (56.0)
	*Mus musculus*	76 (14.8)
	*Arvicola scherman*	64 (12.5)
	*Apodemus sylvaticus*	60 (11.7)
	*Talpa europaea*	14 (2.7)
	*Eliomys quercinus*	8 (1.6)
	*Crocidura russula*	4 (0.8)
Age	Adult	353 (68.7)
	Young	47 (9.1)
	Unknown	114 (22.2)
Sex	Female	265 (51.6)
	Male	208 (40.5)
	Unknown	41 (8.0)
Habitat	Farm (small ruminants)	43 (8.4)
	Wild	349 (67.9)
	Urban	122 (23.7)
Sampling period	2012–2014	92 (17.9)
	2018–2021	136 (26.5)
	2022–2023	263 (51.2)
	Unknown	23 (4.5)
Sampling region	North	381 (74.1)
	Centre	96 (18.7)
	South	37 (7.2)

### Molecular analyses

RNA from liver tissue of all specimens was extracted by the RNeasy mini kit (QIAGEN, Hilden, Germany) using automated procedures (QIAcube, QIAGEN, Hilden, Germany). We screened the presence of ratHEV-C1 RNA by using two previously published real-time RT-qPCR (CFX Connect instrument, Bio-Rad, CA, USA) (Additional files 1 and 2) [4, 13]. Positive samples were then sequenced using nested RT-PCRs (Additional files 1 and 2). Whenever possible, in these infected individuals, the presence of the virus was evaluated in faeces in order to assess the potential excretion of ratHEV-C1. Additionally, ratHEV-C1 was also screened in uninfected individuals. For that, viral RNA was evaluated following the same RT-qPCR protocol described above but extracting the RNA using the IndiSpin Pathogen Kit (Indical Bioscience). More detailed information regarding molecular analyses can be found in the Additional file 1.

### Outcome

The outcome variable was ratHEV-C1 infection, defined as an individual exhibiting detectable viral RNA by either of the RT-qPCR used in liver tissue and confirmed by sequencing.

### Statistical and phylogenetic analyses

The frequency of ratHEV-C1 infection was calculated by dividing the number of ratHEV infected animals by the total number of specimens tested, using two-sided exact binomial 95% confidence intervals (95% CI).

**Figure 1:**
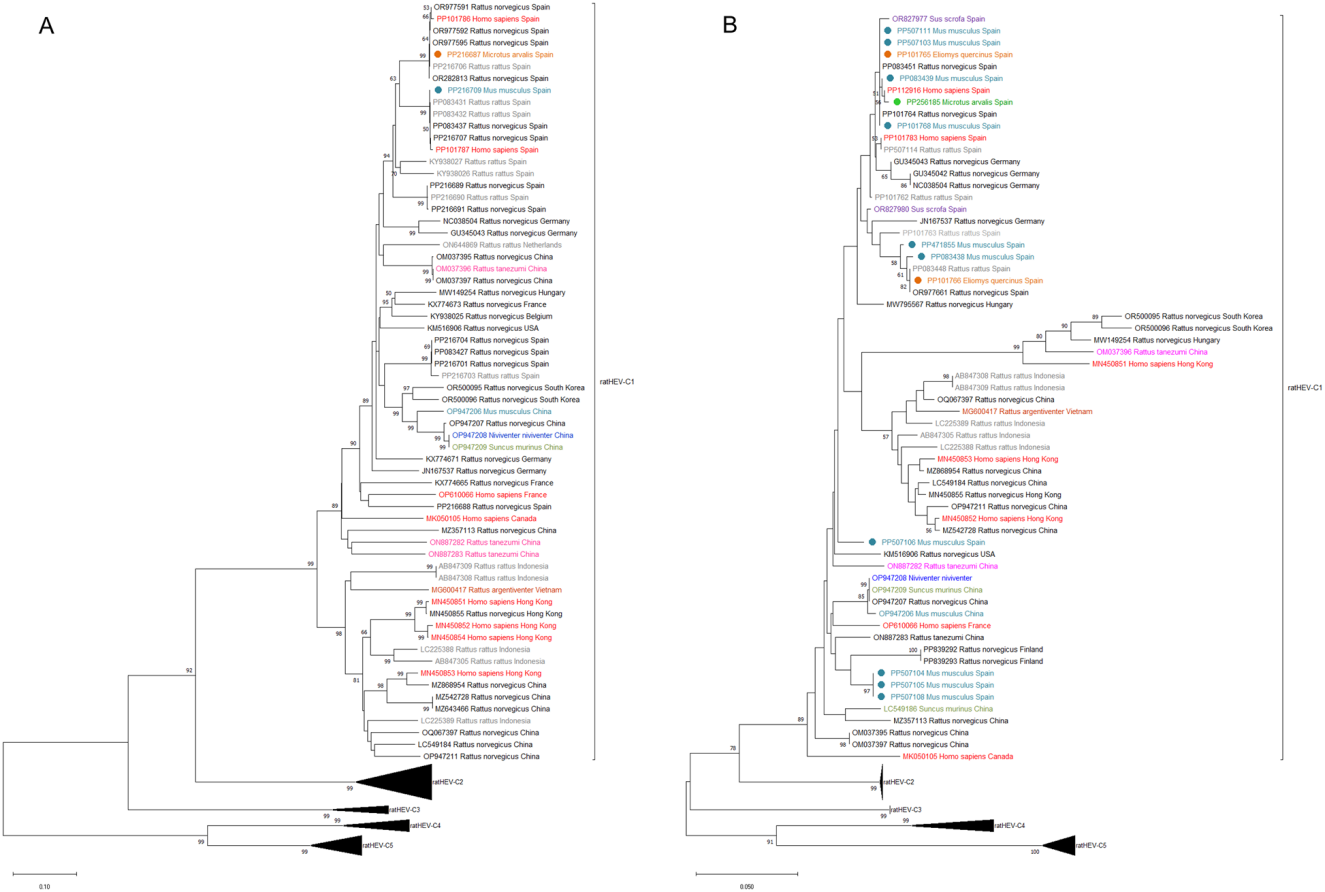
**Phylogenetic tree constructed using the Neighbor Joining method (1000 replicates) with seqPCR-1 (A) or seqPCR-2 and 3 (B).** The tree is drawn to scale, with branch lengths in the same units as those of the evolutionary distances used to infer the phylogenetic tree. Only bootstrap values higher than 50 are shown. Circles indicate the sequences obtained in the present study, and each color represents a different animal species.

Consensus sequences were obtained using SeqMan Software NGen^®^ Version 12.0 (DNASTAR.Madison, WI, USA). ratHEV assignment was performed using the HEVnet genotyping tool [14] and confirmed by BLAST analysis. Phylogenetic trees of each viral region amplified, such as the RNA-dependent RNA polymerase with seqPCR-1 (Figure 1A) and methyltransferase with seqPCR-2 and 3 (Figure 1B), were constructed with the neighbour joining method by the online MAFFT service (Version 10) using the bootstrap method (with 1000 replicates). For this, complete Rocahepevirus sequences from Wu et al. [1] and other representative ratHEV-C1 strains were included. These analyses involved 823 and 139 nt (position 4135–4958 and 259–397, respectively, using GU345042 as reference) from 82 sequences.

## Results

### Study cohort

A total of 514 small mammals from seven species were studied. The greater white-toothed shrew (*Crocidura russula*) (4, 0.8%) and the European common mole (*Talpa europaea*) (14, 2.7%), both distributed among natural environments, crop fields and gardens. The garden dormice, *Eliomys quercinus* (8, 1.6%), that usually inhabits both terrestrial and arboreal environments in wild and agricultural areas. Two mice species, the house mice (*Mus musculus*) (76, 14.8%), a commensal species mainly associated with anthropized environments, and the wood mice (*Apodemus silvaticus*) (60, 11.7%), mainly present in areas with good shrub or tree cover. And finally, the common vole (*Microtus arvalis*) (288, 56.0%), and the fossorial water vole (*Arvicola scherman*) (64, 12.5%) that may reach population averages of 500 individuals per hectare and up to 1000 voles/ha during population peaks, being considered serious agricultural pests and a human health hazard [15, 16]. Detailed information about the sampled population, including spatial distribution, is shown in Table 1 and Additional file 5.

### Screening of ratHEV

We confirmed ratHEV-C1 infection in 15 individuals, supposing a frequency of 2.9% (95% CI: 1.8–4.8) (Additional files 3 and 4). We detected ratHEV-C1 in 14.5% (11/76) of house mice, 0.7% (2/288) of common voles and in two out of the eight sampled garden dormice (Table 2). Positive animals were detected in four urban areas, three small ruminant farms and two different wild areas from Northern, Central and Southern Spain. Faeces could be obtained in 13 of the 15 infected animals. Viral RNA was detected in faeces of four individuals, all of them from house mice, supposing a frequency of detection of 36.3% (4/11) within infected individuals of this species. Additionally, faeces could be collected from 287 uninfected animals and viral RNA was found in 2 (0.7%) individuals (two house mice).

**Table 2:** **Frequency of ratHEV-C1 in infected small mammals according to explanatory variables**

Variable	Categories	No. positives/no. total animals	Frequency (%)
Species	*Microtus arvalis*	2/288	0.7
	*Mus musculus*	11/76	14.5
	*Arvicola scherman*	0/64	0
	*Apodemus sylvaticus*	0/60	0
	*Talpa europaea*	0/14	0
	*Eliomys quercinus*	2/8	25.0
	*Crocidura russula*	0/4	0
Age	Adult	9/353	2.6
	Young	2/47	4.3
Sex	Female	10/265	3.8
	Male	3/208	1.4
Habitat	Farm	4/43	9.3
	Wild	2/349	0.6
	Urban	9/122	7.3

### Phylogenetic analyses

GenBank accession numbers of all sequences are presented in Additional file 3. Phylogenetic analyses evidenced a high genetic diversity of ratHEV-C1 sequences with *p* distances ranging from 0 to 0.091. BLAST analyses revealed a homology ranging from 95 to 100% with ratHEV sequences found in patients with acute hepatitis from Spain and in rats from Spain and other countries of Europe. In the phylogenetic trees constructed, our sequences clustered in three different groups, closely related with ratHEV-C1 strains detected in human and other animal species from Spain, Germany and Hungary (Figures 1A and B).

## Discussion

Owing to the emergence of ratHEV-C1 as a public health issue [3, 17], monitoring this zoonotic virus and identifying all potential animal reservoirs are key to better understanding its epidemiology and the risk of transmission to humans. While ratHEV-C1 circulation appeared to be confined to black and Norway rat populations in the continent [12], our detection of ratHEV-C1 strains in three different species of small mammals expands the host range of this virus, confirms that the virus is not restricted to *Rattus* among animal species and highlight the importance of testing liver for ratHEV-C1 screening in these species. The close nucleotide identity of the highly diverse ratHEV-C1 strains obtained from synanthropic small mammals throughout Spain, compared with those previously found in rats and patients with acute hepatitis, reinforces the zoonotic transmission of this virus and points to the ability of ratHEV-C1 to cross species barriers, highlighting the need to extend the evaluation of ratHEV-C1 to all small mammals. Moreover, the detection of the virus in faeces in a noteworthy proportion of infected mice indicates that exposure not only to rat’s droppings but also to those from mice could be a potential source for ratHEV-C1 infection for humans or other animal species that may act as intermediate host. In this respect, recent studies have suggested spillover transmissions of ratHEV-C1 from rodents to pigs, cats and dogs [18–20].

Interestingly, the high prevalence of 14.5% we found in house mice was similar to the 12.4% detected in black and Norway rat populations from different European countries [12]. These three species belong to *Muridae* family and may share habitat and resources [21], so cross-species transmission of ratHEV-C1 seems plausible. This is supported by the close relationship of most sequences found in our study with those previously identified in rats, consistent with recent findings in Asia [4]. In that study, a positivity rate of 6.4% was found in house mice, with ratHEV-C1 sequences clustering closely with those detected in sympatric *Rattus* individuals. However, the high prevalence observed in house mice in our study, together with the high diversity of ratHEV-C1 strains found in this species, may even indicate the possible role of house mice in the epidemiology of ratHEV-C1 in Europe. However, a relatively high Ct values were observed in some individuals. This might be related to low viral loads in the infected animals. However, the successful sequencing of samples with Ct values exceeding 40 challenges this hypothesis. The divergence of our sequences from those previously detected in other countries suggests that the Ct values could instead be due to sensitivity issues in the qPCR assays, potentially caused by mismatches with primers and/or probes. In any case, further studies are warranted to determine the role of house mice, as well as garden dormice and common vole, in the epidemiology of ratHEV in Europe.

In conclusion, our study confirm that ratHEV-C1 is not limited to *Rattus* genus, identifying other rodents’ species as potential host of ratHEV-C1 in Europe. Our results highlight the importance of continued surveillance in animals to fully understand the dynamics of ratHEV-C1 and its impact on public health.

## Supplementary Information

**Additional file 1.** **Study design and sampling.**

**Additional file 2.** **Additional information regarding molecular analyses.**

**Additional file 3.** **Molecular results of infected small mammals for rat hepatitis E virus.**

**Additional file 4.** **Distribution of ratHEV-C1 infected animals by categories within species.**

**Additional file 5.** **Spatial distribution of small mammals sampled in Northern, Central and Southern Spain.** Pie charts indicate the distribution of species sampled in each sampled Autonomous region. The number of individuals sampled per region is indicated in the centre of each pie chart.

## Data Availability

All data generated or analyzed during the study are included in the article. The datasets used and/or analyzed during the present research project are available from the corresponding author upon reasonable request. The obtained ratHEV sequences were submitted to GenBank (accession numbers: PP101765, PP101766, PP256185, PP216687, PP083439, PP507111, PP083438, PP101768, PP471855, PP216709, PP507106, PP507103, PP507105, PP507108, PP507104).
