# Supplementary material for: Occurrence and genetic diversity of the zoonotic rat hepatitis E virus in small mammal species, Spain
Source: Vet Res. 2025 Mar 25;56:68. doi: 10.1186/s13567-025-01492-1 (PMC11938671; doi:10.1186/s13567-025-01492-1)
Supplement: Supplementary file 1 — Additional file 1. Study design and sampling. [file 13567_2025_1492_MOESM1_ESM.docx]

**Study design and sampling**

To calculate the sampling size, it was assumed a prevalence of 50%, which provides the highest sample size in studies in which prevalence is unknow, a 95.0% confidence level (95%CI), and a desired precision of ±5% [22]. Thus, a sample size of 385 animals was obtained. Samples were obtained from tissue banks or from animals trapped in collaboration with ongoing rodent control campaigns conducted by different pest control companies in urban, periurban and farm settings throughout the country. Carcases were frozen at −20 °C and necropsied in the Department of Animal Health of the University of Cordoba, the Institute for Game and Wildlife Research, or the Regional Service of Research and Agroalimentary Development of Asturias. Epidemiological information about each individual (species, age, sex, habitat, sampling date and sampling location) was recorded whenever possible. The age was estimated based on tooth eruption, reproductive status and body size [23].

**References**

22. Thrusfield MV (2001) Veterinary epidemiology, 3^rd^ edn. Blackwell Science Ltd., UK

23. Rodríguez J L (2008) Guía de campo de los Mamíferos Terrestres de España. ISBN: 97884282091
