# Supplementary material for: Occurrence and genetic diversity of the zoonotic rat hepatitis E virus in small mammal species, Spain
Source: Vet Res. 2025 Mar 25;56:68. doi: 10.1186/s13567-025-01492-1 (PMC11938671; doi:10.1186/s13567-025-01492-1)
Supplement: Supplementary file 2 — Additional file 2. Additional information regarding molecular analyses. [file 13567_2025_1492_MOESM2_ESM.docx]

**Molecular analyses**

The primers and probes for molecular assays were supplied by IDT (Integrated DNA Technologies, IA, USA) and purified by the desalting technique, being finally reconstituted at a concentration of 20μM. RT-qPCRs were performed in a CFX Connect instrument (Bio-Rad, CA, USA) using the One Step PrimeScript III RT-PCR Kit (Takara Bio, Shiga, Japan) and carried out in final volumes of 50μl containing 20μl of the One Step PrimeScript III RT-qPCR Mix, 17.75μl of RNase-DNase free water, 0.75μl of forward (20μM), 0.75μl of reverse (20μM), 0.75μl of probe (20μM) and 10μl of RNA. The thermal profile was 25ºC for 10 min, 52ºC for 5 min and 95ºC for 10 sec, followed by 45 cycles of 95ºC for 5 sec and 59ºC (for qPCR-1) or 54ºC (for qPCR-2) for 30 sec. Positive samples were then sequenced following nested RT-PCRs with the One Step PrimeScript III RT-PCR Kit master mix (Takara Bio, Japan) and the premixed 2X solution Taq DNA polymerase, dNTPs and reaction buffer kit (Promega, USA) for the first and second round of PCR, respectively. The first round of nested RT-PCRs were carried out with the same volumes for primers, master mix and RNA as described above but adding 18.5 μl of RNase-DNase free water. The second round was performed with 25μl of master mix, 15μl of RNase-DNase free water, 2.5μl of each primer (20μM each) and 5μl of template. The second PCR products were examined on 1.5% agarose gel stained with RedSafe™ Nucleic Acid Staining solution. Positive samples were purified using the Illustra™ ExoProStar™ 1-Step kit (VWR, USA) and submitted to STAB VIDA for sequencing. The thermal profile for the first round of RT-PCRs was identical to qPCRs except the last temperature, being 58ºC for seqPCR-1 and 55ºC seqPCR-2 and seqPCR-3. The thermocycling conditions for the nested PCR were 95ºC for 2 min, followed by 45 cycles of 95ºC 1 min, 58ºC (for seqPCR-1) or 55ºC (for seqPCR-2 and seqPCR-3) for 1 min and 72ºC for 1 min, with a final extension at 72ºC during 5 min. As positive control in both qPCR and nested RT-PCR, we used RNA from a rodent liver sample previously tested as ratHEV positive in our laboratory (GenBank Accession number: OR282813 and OR977661). Likewise, we used nuclease-free water as negative control in each run of PCR. The primer pairs of each qPCR and nested RT-PCR are shown in the following table.

Table. List of primers and probe used for screening and sequencing ratHEV.

| **ID PCR** | **Reference** | **Target* (pb)**  **(position**)** | **Primers (5'-3')** | | **Probe (5’-3’)** |
| --- | --- | --- | --- | --- | --- |
|  |  |  | **Forward**  **(position*)** | **Reverse**  **(position*)** |  |
| qPCR-1 | [4] | Met (69) (146-214) | CTTGTTGAGCTYTTCTCCCCT | CTGTACCGGATGCGACCAA | HEX- TGCAGCTTGTCTTTGARCCC-IABkFQ |
| qPCR-2 | [24] | Met (173) (36-208) | CCACGGGGGTTAATACTGC | CGGATGCGACCAAGAAACAG | 6FAM-CGGCTACCGCCTTTGCTAATGC-BBQ |
| seqPCR-1 | [13] | RdRp  (880)  (4150-5029) | 1^st^ PCR: CTTGGTTYAGGGCCATAGAG | 1^st^ PCR: CAGCAGCGGCACGAACAGCA |  |
|  |  |  | 2^nd^ PCR: TTYAGGGCCATAGAGAAGGC | 2^nd^ PCR: ACAGCAAAAGCACGAGCACG |  |
| seqPCR-2 | - | Met (287)  (164-450) | 1^st^ PCR: TTTGCTAATGCTCAGGTGGT | 1^st^ PCR: CATDCCATGAGCACGCAT |  |
|  |  |  | 2^nd^ PCR: CCTYTGCAGCTTGTCTTTGA | 2^nd^ PCR: GTGCAAAAGGAAAGATCAG |  |
| seqPCR-3 | [9] | Met (243)  (189-450) | 1^st^ PCR: CCTYTGCAGCTTGTCTTTGA | 1^st^ PCR: CATDCCATGAGCACGCAT |  |
|  |  |  | 2^nd^ PCR: CTGTTTCTTGGTCGCATCCG | 2^nd^ PCR: GTGCAAAAGGAAAGATCAG |  |
| * Met: Methyltransferase, RdRp: RNA-dependent RNA polymerase  **Genome positions respect to the reference ratHEV sequence under the GenBank Accession number GU345042 | | | | | |

**References**

24. Suparyatmo JB, Andayani IGAS, Takahashi M, Ohnishi H, Jirintai S, Nagashima S, Nishizaaw T, Okamoto H (2014) Marked genomic heterogeneity of rat hepatitis E virus strains in Indonesia demonstrated on a full-length genome analysis. Virus Res 179:102–112
