# Supplementary material for: Occurrence and genetic diversity of the zoonotic rat hepatitis E virus in small mammal species, Spain
Source: Vet Res. 2025 Mar 25;56:68. doi: 10.1186/s13567-025-01492-1 (PMC11938671; doi:10.1186/s13567-025-01492-1)
Supplement: Supplementary file 3 — Additional file 3. Molecular results of infected small mammals for rat hepatitis E virus. [file 13567_2025_1492_MOESM3_ESM.docx]

**Additional file 3 Molecular results of infected small mammals for rat hepatitis E virus.**

| **ID** | **Species** | **qPCR-1** | **Ct qPCR-1** | **qPCR-2** | **Ct qPCR-2** | **GenBank Accesion Number (seqPCR)** |
| --- | --- | --- | --- | --- | --- | --- |
| 1 | *Eliomys quercinus* | - |  | + | 42.15 | PP101765 (seqPCR-2) |
| 2 | *Eliomys quercinus* | + | 32.01 | + | 38.06 | PP101766 (seqPCR-2) |
| 3 | *Microtus arvalis* | + | 34.69 | - |  | PP256185 (seqPCR-2) |
| 4 | *Microtus arvalis* | + | 34.00 | - |  | PP216687 (seqPCR-1) |
| 5 | *Mus musculus* | + | 37.50 | - |  | PP083439 (seqPCR-2) |
| 6 | *Mus musculus* | - |  | + | 37.18 | PP507111 (seqPCR-3) |
| 7 | *Mus musculus* | + | 37.08 | - |  | PP083438 (seqPCR-2) |
| 8 | *Mus musculus* | + | 37.73 | - |  | PP101768 (seqPCR-3) |
| 9 | *Mus musculus* | + | 33.02 | + | 36.20 | PP471855 (seqPCR-2) |
| 10 | *Mus musculus* | + | 38.30 | + | 37.97 | PP216709 (seqPCR-1) |
| 11 | *Mus musculus** | + | 26.54 | + | 36.8 | PP507106 (seqPCR-3) |
| 12 | *Mus musculus** | - |  | + | 41.08 | PP507103 (seqPCR-3) |
| 13 | *Mus musculus** | + | 33.75 | - |  | PP507105 (seqPCR-3) |
| 14 | *Mus musculus* | + | 34.34 | + | 36.71 | PP507108 (seqPCR-3) |
| 15 | *Mus musculus** | + | 37.50 | + | 39.32 | PP507104 (seqPCR-3) |
| *These animals tested also positive for ratHEV in faeces. Ct values from faeces were: ID11: 28.9 (qPCR-1) and 30.2 (qPCR-2), ID12: 37.2 (qPCR-1), ID13: 40.5 (qPCR-1) and ID15: 37.1 (qPCR-1) | | | | | | |
