# Supplementary material for: Occurrence and genetic diversity of the zoonotic rat hepatitis E virus in small mammal species, Spain
Source: Vet Res. 2025 Mar 25;56:68. doi: 10.1186/s13567-025-01492-1 (PMC11938671; doi:10.1186/s13567-025-01492-1)
Supplement: Supplementary file 4 — Additional file 4. Distribution of ratHEV-C1 infected animals by categories within species. [file 13567_2025_1492_MOESM4_ESM.docx]

**Additional file 4 Distribution of ratHEV-C1 infected animals by categories within species.**

| Variable | Category | Species | Positive | Total |
| --- | --- | --- | --- | --- |
| Region | North | *Apodemus sylvaticus* | 0 | 8 |
|  |  | *Arvicola scherman* | 0 | 64 |
|  |  | *Crocidura russula* | 0 | 4 |
|  |  | *Eliomys quercinus* | 0 | 0 |
|  |  | *Mus musculus* | 0 | 4 |
|  |  | *Talpa europaea* | 0 | 14 |
|  |  | *Microtus arvalis* | 2 | 287 |
|  | South | *Apodemus sylvaticus* | 0 | 0 |
|  |  | *Arvicola scherman* | 0 | 0 |
|  |  | *Crocidura russula* | 0 | 0 |
|  |  | *Eliomys quercinus* | 2 | 8 |
|  |  | *Mus musculus* | 5 | 29 |
|  |  | *Talpa europaea* | 0 | 0 |
|  |  | *Microtus arvalis* | 0 | 0 |
|  | Centre | *Apodemus sylvaticus* | 0 | 52 |
|  |  | *Arvicola scherman* | 0 | 0 |
|  |  | *Crocidura russula* | 0 | 0 |
|  |  | *Eliomys quercinus* | 0 | 0 |
|  |  | *Mus musculus* | 6 | 42 |
|  |  | *Talpa europaea* | 0 | 0 |
|  |  | *Microtus arvalis* | 0 | 1 |
| Sex | Female | *Apodemus sylvaticus* | 0 | 13 |
|  |  | *Arvicola scherman* | 0 | 32 |
|  |  | *Crocidura russula* | 0 | 1 |
|  |  | *Eliomys quercinus* | 2 | 8 |
|  |  | *Mus musculus* | 7 | 39 |
|  |  | *Talpa europaea* | 0 | 1 |
|  |  | *Microtus arvalis* | 1 | 170 |
|  | Male | *Apodemus sylvaticus* | 0 | 24 |
|  |  | *Arvicola scherman* | 0 | 31 |
|  |  | *Crocidura russula* | 0 | 2 |
|  |  | *Eliomys quercinus* | 0 | 0 |
|  |  | *Mus musculus* | 2 | 22 |
|  |  | *Talpa europaea* | 0 | 13 |
|  |  | *Microtus arvalis* | 1 | 116 |
| Age | Adult | *Apodemus sylvaticus* | 0 | 17 |
|  |  | *Arvicola scherman* | 0 | 61 |
|  |  | *Crocidura russula* | 0 | 1 |
|  |  | *Eliomys quercinus* | 0 | 1 |
|  |  | *Mus musculus* | 9 | 68 |
|  |  | *Talpa europaea* | 0 | 14 |
|  |  | *Microtus arvalis* | 0 | 190 |
|  | Young | *Apodemus sylvaticus* | 0 | 5 |
|  |  | *Arvicola scherman* | 0 | 3 |
|  |  | *Crocidura russula* | 0 | 0 |
|  |  | *Eliomys quercinus* | 2 | 7 |
|  |  | *Mus musculus* | 2 | 7 |
|  |  | *Talpa europaea* | 0 | 0 |
|  |  | *Microtus arvalis* | 0 | 25 |
| Habitat | Farm (small ruminants) | *Apodemus sylvaticus* | 0 | 0 |
|  |  | *Arvicola scherman* | 0 | 0 |
|  |  | *Crocidura russula* | 0 | 0 |
|  |  | *Eliomys quercinus* | 0 | 0 |
|  |  | *Mus musculus* | 4 | 43 |
|  |  | *Talpa europaea* | 0 | 0 |
|  |  | *Microtus arvalis* | 0 | 0 |
|  | Urban | *Apodemus sylvaticus* | 0 | 4 |
|  |  | *Arvicola scherman* | 0 | 64 |
|  |  | *Crocidura russula* | 0 | 1 |
|  |  | *Eliomys quercinus* | 2 | 8 |
|  |  | *Mus musculus* | 7 | 28 |
|  |  | *Talpa europaea* | 0 | 14 |
|  |  | *Microtus arvalis* | 0 | 2 |
|  | Wild | *Apodemus sylvaticus* | 0 | 56 |
|  |  | *Arvicola scherman* | 0 | 0 |
|  |  | *Crocidura russula* | 0 | 3 |
|  |  | *Eliomys quercinus* | 0 | 0 |
|  |  | *Mus musculus* | 0 | 4 |
|  |  | *Talpa europaea* | 0 | 0 |
|  |  | *Microtus arvalis* | 2 | 286 |
