# Supplementary material for: Occurrence and genetic diversity of the zoonotic rat hepatitis E virus in small mammal species, Spain
Source: Vet Res. 2025 Mar 25;56:68. doi: 10.1186/s13567-025-01492-1 (PMC11938671; doi:10.1186/s13567-025-01492-1)
Supplement: Supplementary file 5 — Additional file 5. Spatial distribution of small mammals sampled in Northern, Central and Southern Spain. Pie charts indicate the distribution of species sampled in each sampled Autonomous region. The number of individuals sampled per region is indicated in the centre of each pie chart. [file 13567_2025_1492_MOESM5_ESM.docx]

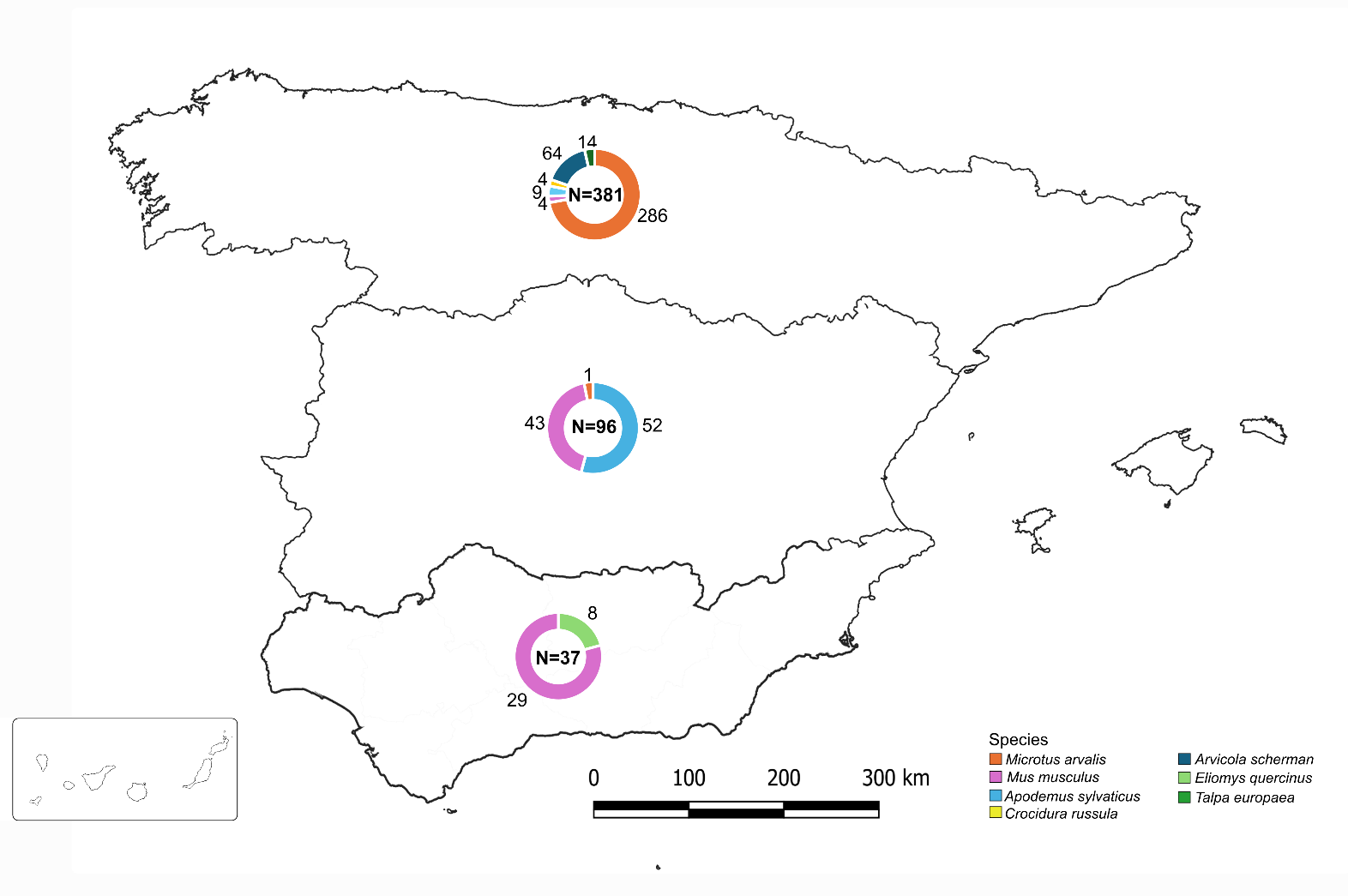
**Figure.** Spatial distribution of small mammals sampled in Northern, Central and Southern Spain. Pie charts indicate the distribution of species sampled in each sampled Autonomous region. The number of individuals sampled per region is indicated in the centre of each pie chart.
